# Transcranial Direct Current Stimulation in Stroke Rehabilitation: A Review of Recent Advancements

**DOI:** 10.1155/2013/170256

**Published:** 2013-02-27

**Authors:** Andrea Gomez Palacio Schjetnan, Jamshid Faraji, Gerlinde A. Metz, Masami Tatsuno, Artur Luczak

**Affiliations:** Canadian Centre for Behavioural Neuroscience, Department of Neuroscience, University of Lethbridge, 4401 University Drive, Lethbridge, AB, Canada T1K 3M4

## Abstract

Transcranial direct current stimulation (tDCS) is a promising technique to treat a wide range of neurological conditions including stroke. The pathological processes following stroke may provide an exemplary system to investigate how tDCS promotes neuronal plasticity and functional recovery. Changes in synaptic function after stroke, such as reduced excitability, formation of aberrant connections, and deregulated plastic modifications, have been postulated to impede recovery from stroke. However, if tDCS could counteract these negative changes by influencing the system's neurophysiology, it would contribute to the formation of functionally meaningful connections and the maintenance of existing pathways. This paper is aimed at providing a review of underlying mechanisms of tDCS and its application to stroke. In addition, to maximize the effectiveness of tDCS in stroke rehabilitation, future research needs to determine the optimal stimulation protocols and parameters. We discuss how stimulation parameters could be optimized based on electrophysiological activity. In particular, we propose that cortical synchrony may represent a biomarker of tDCS efficacy to indicate communication between affected areas. Understanding the mechanisms by which tDCS affects the neural substrate after stroke and finding ways to optimize tDCS for each patient are key to effective rehabilitation approaches.

## 1. Introduction 

Poststroke consequences including sensorimotor and cognitive impairments impose a stressful situation and a great burden to the victims, their families, and the society. Indeed, stroke is one of the leading causes of adult disability in the western world [1]. Among extensive efforts devoted to the search for more effective rehabilitation therapies of stroke, the idea of using electricity can be traced back almost a century ago (as noted by Priori [2]). After diminished interest due to mixed results, recent studies with promising results regained the interest in the application of mild electrical currents to the brain as a potential therapy for neurological disorders [2]. Research by Priori [2, 3] and Nitsche and colleagues [4–6] led to the development of a technique consisting of the application of weak electrical currents through the scalp, which is now called transcranial direct current stimulation (tDCS). Recent findings suggest that tDCS may be beneficial in a wide range of disorders such as epilepsy [7, 8], Parkinson's disease [9–11], chronic pain [12–14], depression [15], drug cravings [16], pain conditions such as fibromyalgia [17–19], and traumatic spinal cord injuries [12, 20, 21]. Over the past few years, the potential therapeutic benefit of tDCS for improvement of cerebral function after stroke has also been reported [22–28]. Nevertheless, more evidence is needed in order to consider tDCS as a standard therapeutic technique to help patients with stroke and other brain disorders.

The modulation of cortical excitability by tDCS has gained particular interest because of its beneficial neurorehabilitative effects after stroke [29]. However, neither the detailed mechanisms of how tDCS facilitates recovery from stroke nor optimal parameters of stimulation are well understood yet, thus limiting the tDCS application to stroke patients [30]. Elucidation of the physiological mechanisms of tDCS and the optimization toward the need for stroke rehabilitation would be crucial in successful use of this therapy [31]. This paper will, therefore, focus on the physiological effects of tDCS and their implications in stroke rehabilitation.

After stroke, considerable modifications in synaptic organization and plasticity take place. They may account for some of the spontaneous recovery in the loss of sensation, movement, or cognition following stroke [32]. It has been suggested that increased general cortical excitability as well as modifications in synaptic plasticity such as LTP-like modulation, increments in calcium currents, and activation of neurotrophic factors in the affected hemisphere are relevant mechanisms for stroke recovery [32]. However, it should be noted that these effects might be region-specific and could be related to the orientation of the stimulated fibers. In addition, different protocols of stimulation, electrode position, and current polarization make it difficult to determine the appropriate parameters for stroke rehabilitation [31]. Due to the significant reorganization of neuronal connections after stroke, it would also be necessary to evaluate the effects of tDCS as a function of post-infarct interval. 

This paper will summarize recent findings that support tDCS as a suitable complementary rehabilitative technique to promote stroke recovery. We will also discuss the possibility of determining stimulation parameters based on electrophysiological activity. We propose that if stimulation protocols can be adjusted to individual needs, tDCS would become a more effective therapy to support recovery from stroke.

## 2. General Considerations of the Technique

**Figure 1:**
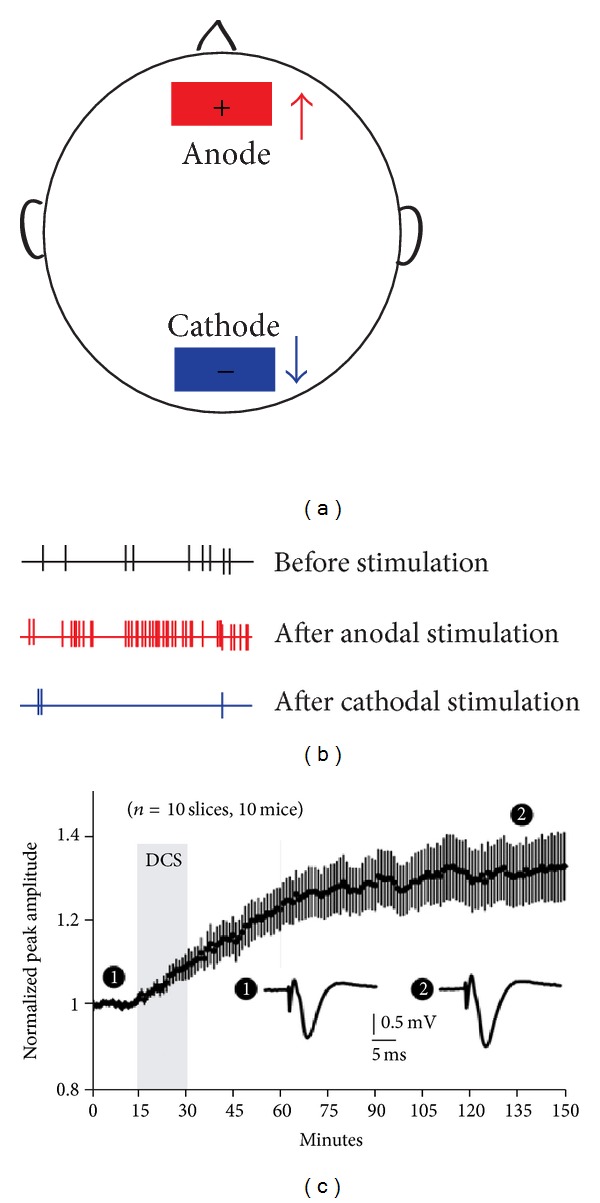
Physiological effects of tDCS. (a) Illustration of the typical placement of the anode (red square) and cathode (blue square) during stimulation of the primary motor cortex. The direction of stimulation causes differential effects on neuronal activation and plasticity. (b) Illustration of anodal (red) and cathodal (blue) transcranial direct current stimulation on spike activity in animals (modified from [39]). Anodal stimulation increased subsequent spike activity by lowering the membrane potential, whereas cathodal stimulation reduced subsequent spike activity in the stimulated area by increasing the membrane potential. (c) DCS promotes LTP in motor cortical slices. The sample of fEPSPs showing a 2-hour time course after DCS (vertical gray line) (from [40]).

A detailed description of the methodological procedure has been published in numerous previous publications [2–6, 30, 33–36]. Generally, tDCS protocols utilize two surface electrodes, one serving as the anode and the other one as the cathode or reference, although other configurations have been also reported [37, 38]. The position of the electrodes appears to be critical for the spatial distribution and direction of the flow of the current which may determine the effectiveness of the stimulation. It is generally agreed that anodal tDCS has an excitatory effect on the local cerebral cortex by depolarizing neurons, while the converse applies to the cathode through the process of hyperpolarization (Figure 1(a)). Typically these electrodes have relatively large surfaces of 20–35 mm^2^ that limit the focus of stimulation. On the other hand, the large surface allows the use of low current densities, which constitutes one of the critical parameters for patient safety [5].

It has also been widely accepted that the surface area of the electrodes determines the outcome of transcranial stimulation [41]. For example, increasing the surface area of the reference electrode and reducing the surface of the stimulation electrode allow for more focal treatment effects [42]. Increasing the distance between the electrodes has been shown to enhance the current flow into the brain and the depth of the current density [37]. Maintaining low current densities is important to prevent patient's discomfort and allows application of tDCS for long periods of time [4]. A direct current of 1-2 mA has been generally applied for a timespan ranging between 8 and 30 min. Approximately 45% of this range of the current delivered to the skull reaches the surface of the cortex [6]. 

In order to evaluate the nureophysiological effects of tDCS in stroke rehabilitation, clinical and preclinical studies need to be accompanied by animal model studies. So far, there has been proposed multiple approaches to study electrophysiological effects of direct current stimulation in animals [40, 43–49]. In this paper, we included the results of those studies in animal models, but it is important to note that some of the animal studies are not strictly comparable to the human tDCS method. For example, to achieve more localized effects, in animal studies electrodes may be placed on the top of the dura mater or intracortically, thus making it more difficult to directly relate to human tDCS. 

In summary, tDCS has been shown to be easy to apply, inexpensive, and portable. It can be applied simultaneously with other rehabilitation therapies and can potentially affect a range of neuronal networks. 

## 3. Physiological Mechanisms of Action: Implications for Stroke Recovery

### 3.1. Effects of tDCS on Cortical Excitability

Oxygen deprivation occurring during stroke rapidly reduces the normal functioning of neurons and makes it impossible to maintain their normal transmembrane ionic gradients. This results in an ion and water imbalance that initiates apoptotic and necrotic cell death cascades. It ultimately leads to loss or impairment of sensory, motor or cognitive function depending on the location of the stroke [50]. In the first few days or weeks after stroke, the normal patterns of synaptic excitatory activity are disrupted in the areas surrounding the infarct [51–54] and in remote areas functionally connected to the infarct site [39, 55].

The reduction in neuronal activation is proposed to be induced by the loss of inputs from adjacent tissue that is affected by the infarct, edema, reduced cerebral blood flow, and metabolic depression [32]. The plastic rearrangements after brain damage, possibly attempting to regain function, reset the level of activity in these neurons. Following the first week of stroke, there is generally an exacerbated excitability in the penumbra [56–58] as well as in areas remote to the lesion [55]. The latter include the intact contralesional hemisphere [59–61]. For example, hyperexcitability and associated reduction of GABA_A_ receptor expression in the surround of photothrombotic infarcts (2-3 mm apart from the lesion) may begin within a week after lesion and continue up to four months in rats [57]. Although such increased excitability has aberrant properties, it is believed to be responsible for the majority of spontaneous recovery following stroke. Indeed, it was shown that increased excitability in surviving neurons might lead to the transient appearance of patterned, low-frequency spontaneous activity that contributes to a permissive environment for axonal sprouting in rat focal ischemia models [62]. Interestingly, this low-frequency activity was observed only 1–3 days after stroke, suggesting that it has a critical period. Therefore, it has been proposed that increased neuronal activity in this critical period following stroke might positively influence the recovery from stroke. 

The detailed mechanisms of tDCS have yet to be elucidated. Several studies reported that anodal stimulation increases the spontaneous firing rate and the excitability of cortical neurons by depolarizing the membranes [44, 46, 48, 63–66]. By contrast, cathodal stimulation leads to neuronal hyperpolarization resulting in a decrease of the neuronal firing rate and excitability [63, 65] (Figure 1(b)). This pattern of activity was first shown in animals receiving stimulation via epidural or intracerebral electrodes [63–65]. It has also been reported that the direction of cortical modulation depends not only on the polarity of electrodes but also on the type and the spatial orientation of neurons as well as the stimulation intensity. For example, under certain parameters the neurons in the deeper layers of the neocortex can be activated by cathodal and inhibited by anodal stimulation, possibly as a result of the inversion of current flow associated with the neuron's spatial orientation [64]. It has been reported that high current intensities are required to activate pyramidal cells, whereas weak stimulation is enough to activate nonpyramidal neurons [65]. 

Studies in humans are consistent with these effects [6, 34, 42, 67–74]. Nitsche and Paulus [6] demonstrated that excitation can be induced by anodal tDCS, and inhibition can be achieved by cathodal tDCS. These researchers also reported prolonged after effects of tDCS (up to 90 min) in human motor cortex that depends on the duration of stimulation and current intensity [6, 34, 67, 68, 72, 75–81]. These effects on cortical inhibition suggest that tDCS modulates the excitability of both inhibitory interneurons as well as excitatory neurons [41]. 

Modifications in synaptic excitability are relevant to stroke rehabilitation because this process would attenuate the shift of cortical topographic representation that usually occurs after focal ischemic lesions [82]. Teskey and colleagues showed that epidural currents at a frequency of 50 Hz or higher are associated with long-term improvements in skilled movement and potentiation of the polysynaptic component of cortical evoked potentials *in vitro *and *in vivo* [44, 83, 84]. It is important to note that the intermittent stimulation sessions that were repeated for several days were required before a significant potentiation could be observed.

The effects of cathodal and anodal stimulation on neural membrane may be explained by a number of possible mechanisms including local changes in ionic concentrations, alterations in transmembrane proteins, and electrolysis-related changes in hydrogen ion concentration induced by exposure to a constant electric field [41]. Pharmacological studies offer some verification for these mechanisms. It has been reported, for example, that sodium and calcium channel blockers eliminate both the immediate and longer term effects of anodal stimulation [85]. Blocking N-methyl-D-aspartate (NMDA) receptors for glutamate has been shown to prevent the long-term effects of tDCS, regardless of its direction [85–87]. It was also found that anodal stimulation has a significant positive effect on I-wave facilitation. I-waves are modulated by GABAergic drugs and ketamine, an NMDA-receptor antagonist, but not by ion channel blockers [88]. Thus, this observation suggests effects of anodal stimulation on inhibitory synaptic pathways [41]. 

### 3.2. tDCS and Synaptic Plasticity

Recovery is usually measured as a behavioural change following injury or trauma. It is assumed that these behavioural changes will be correlated with plastic changes in cerebral organization. However, the coexistence of multiple types of cellular and network changes after stroke makes it difficult to search for causal brain-behaviour associations [89].

In stroke survivors, a dynamic neuroplastic process is initiated by an increase in perilesional excitability mediated by excitatory neurotransmitters in the acute and subacute phase. This is followed by a chronic phase that consists of a more complex series of modifications of intracortical and interhemispheric inhibition, which either facilitate or hinder spontaneous recovery [35]. New structural and functional circuits can be formed through reorganization of related cortical regions. Reorganization can occur by remapping representations of lesional areas onto nonlesional cortex, either in the perilesional areas or in the contralesional hemisphere. Many fundamental mechanisms of stroke recovery are based on both structural and functional changes in brain circuits that resemble those commonly observed in neuronal development [32].

It is important to note that neuroplasticity after a stroke might not always facilitate recovery. Plasticity may also have maladaptive consequences, leading to excitability changes or rewiring patterns that interfere with recovery. Aberrant activation patterns seen in brain imaging studies and excitability shifts in transcranial magnetic stimulation (TMS) studies may be indicative of this maladaptation.

It is also possible that physiological LTP is partially responsible for topographic motor map reorganization and that this is facilitated by surface electrical stimulation. Nudo and collaborators [47] showed that a repeated application of very low-intensity electrical stimulation to the motor cortex induced changes in movement representations. A more recent report showed that LTP of the polysynaptic component of the neocortical evoked potential resulted in an expansion of the caudal forelimb area [82]. These findings support the notion that the neural mechanisms underlying the reorganization of motor maps may be based, in part, upon enhanced synaptic strengthening of the horizontal connections. In this regard, Plautz et al. [90] and Kleim et al. [91] showed that cortical stimulation can reorganize movement representations to peri-infarct areas in primates and rats after ischemic lesions to their motor cortices. The importance of contralesional (ipsilateral to the moving hand) activation during motor tasks involving the recovering hand or arm is not clear. The effects seem to range from neutral or positive consequence such as adaptive neuroplastic process to negative maladaption that may interfere with recovery. 

Although there is some indication that tDCS itself can result in lasting changes in neural responses, there is substantial evidence that when combined with tetanic stimulation or a sensorimotor task, long-lasting changes in synaptic strength can be induced [40, 46, 63, 92, 93]. Delivery of a tetanic current can have dramatic and lasting effects on the amplitude of event-related potentials. It has been demonstrated that tDCS changes the spontaneous firing rate of cortical neurons in rats [63, 64, 66]. Plasticity may be induced by spike-timing-dependent plasticity-like interactions between DCS-induced neuronal activity and discrete stimulation. Stimulation could be effective in the form of electrical pulses in *ex vivo* slices [40], as repetitive TMS [94] or as sensory stimulation resulting from participation in a rehabilitative task in humans [92]. The fact that low frequencies, which would normally induce long-term depression (LTD), cause LTP when paired with DCS suggests that postsynaptic cells are in a permissive state for LTP as a result of DCS. Similarly, clamping cells in a depolarized state also result in low-frequency-induced LTP [95]. 

One of the most relevant evidence of tDCS-induced long-lasting change comes from a study performed by Fritsch et al. [40]. These authors applied anodal tDCS to slices of mouse primary motor cortex and showed that it elicits only a short-lasting potentiation. However, if tDCS was coupled with simultaneous low frequency stimulation (0.1 Hz), a long-lasting LTP is obtained (Figure 1(c)) [95]. Furthermore, LTP was specific to polarity (no effects with cathodal DCS) and is related to the frequency of simultaneous stimulation (lower and higher frequencies do not produce LTP). Finally, LTP induction was dependent on NMDA receptor activation and required activity-dependent brain-derived neurotrophic factor (BDNF) secretion [40]. Other factors related to tDCS induced long-term synaptic modifications include protein synthesis by sustained excitability [96]. This may extend the time window in which morphological modifications and synapse formation may occur [97]. In summary, these studies show that both beneficial and maladaptive effects of tDCS are closely related to synaptic plasticity. With better mechanistic understanding, tDCS may provide a useful tool for clinicians to modulate synaptic plasticity and ameliorate functional loss in stroke [98].

### 3.3. tDCS and Neurotrophins

The production and release of neural growth factors after stroke generate a permissive environment for neuronal regeneration in the perilesional cortex [32]. These proteins may be responsible for a large part of synaptic modifications that facilitate recovery after stroke. It is known that tDCS facilitates the release of BDNF, which modulates the induction of NMDAR-dependent LTP through the TrkB receptor [40]. Slices taken from genetically manipulated mice that do not express BDNF or in which the TRkB receptor has been deleted fail to show tDCS-induced LTP [98]. Consequently, mice which do not express BDNF have significant deficits in motor learning [40]. A common polymorphism (Val66Met), found in about 30% of the human population, is associated with reduced BDNF concentrations in the synaptic cleft and with impaired motor learning in animal models [98]. This finding suggests that a significant minority of the human population may suffer from a mild impediment in motor skill acquisition [98–100]. Indeed, humans possessing this polymorphism show a reduction in motor cortical plasticity induced by tDCS/rTMS applied to motor cortex [94, 101]. Taken together, these findings indicate that the effect of tDCS may be facilitated by changing neurotrophic factor support.

## 4. Interhemispheric Competition and the Application of tDCS after Stroke

Cerebral stroke is functionally characterized by alterations of interhemispheric interactions in which neuronal activity in the unaffected hemisphere increases while activity in the affected hemisphere decreases [35, 102–104]. This leads to maladaptive neural activation patterns that are mainly caused by imbalance of interhemispheric inhibition. In other words, the imbalance caused by the unaffected hemisphere imposes a more active inhibitory transcallosal signal to the affected hemisphere. Accordingly, this maladaptive phenomenon impedes functional recovery following stroke. For example, the extent of transcallosal inhibition from the contralateral (unaffected) hemisphere of the ipsilateral (affected) hemisphere is positively correlated with the severity of motor deficits of the affected hand [102].

**Figure 2:**
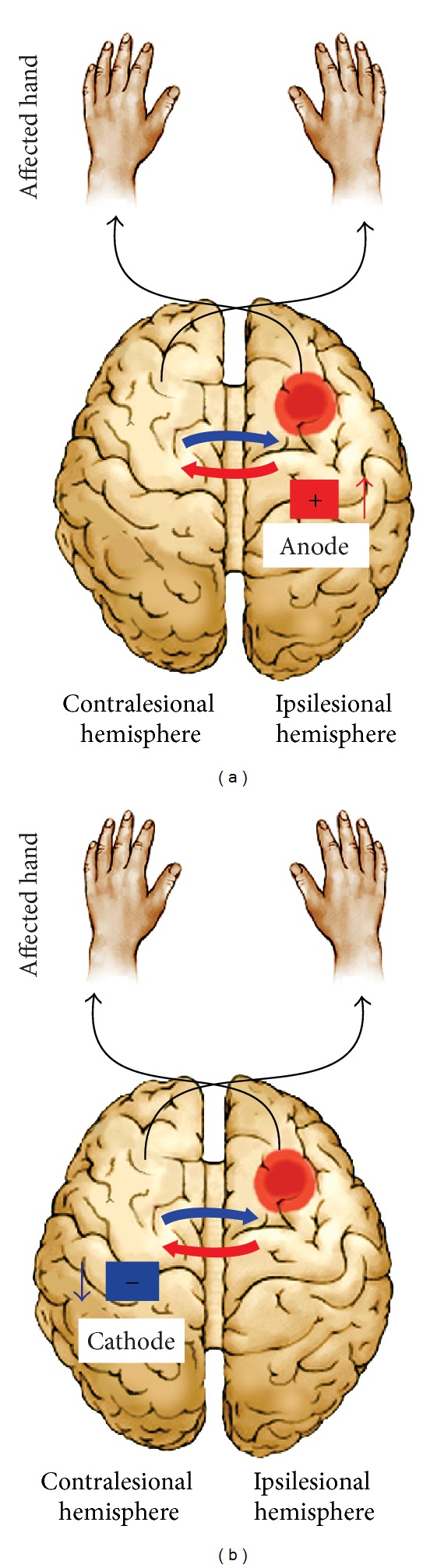
Interhemispheric competition following a stroke. The model suggests that the contralesional (unaffected) motor region exerts an excessive inhibitory influence on the ipsilesional (affected) motor cortex which might limitpoststroke motor recovery. The model provides a hypothetical framework for developing therapeutic strategies. (a) Upregulation of neural excitability of the intact regions of the ipsilesional (affected) motor cortex by anodal tDCS. (b) Downregulation of excitability of the contralesional (unaffected) motor cortex by cathodal tDCS.

To resolve these issues, tDCS can be applied focally to balance the level of hemispheric excitability [105] and modulate spontaneous neuronal activity in a polarity-dependent manner [46]. Excitability of a specific region can be increased by anodal stimulation or decreased by cathodal stimulation [63]. Unlike TMS, which is both a neurostimulatory and a neuromodulatory application, tDCS seems to provide a neuromodulatory intervention only [103]. The electrical currents in tDCS facilitate modulation of the neuronal resting membrane potentials. Anodal tDCS causes subthreshold depolarization and enhances the excitability of the affected hemisphere. Cathodal tDCS, on the other hand, induces hyperpolarization and reduces the excitability of the unaffected hemisphere [6]. According to this model, inhibition of the unaffected hemisphere by cathodal tDCS or excitation of the affected hemisphere by anodal tDCS may normalize the poststroke bihemispheric imbalance of transcallosal inhibition (Figure 2) [33].

Although some studies failed to report therapeutic effects of tDCS [27, 106], investigations mostly showed that tDCS represents a valuable procedure to relieve poststroke symptoms. These reports indicate that tDCS needs to be individually tailored for greater success. They also suggest that the therapeutic effectiveness of tDCS depends upon a number of variables such as lesion size and location, the type and the extent of the functional impairments, and the time interval after stroke when tDCS is applied. The frequency and duration of tDCS application are critical factors for restoration of equilibrium in disrupted networks, improved structural plasticity, and functional recovery [6, 44, 107]. Whether tDCS is effective at chronic intervals after stroke has not yet been completely understood, but one study using tDCS at 3.7 ± 1.1 year after stroke reported a beneficial effect [108]. It should also be noted that both immediate and chronic alterations seem to depend on the neuroplastic capacity of the brain as was indicated by studies in rodent models [109] and primates [110].

Neuroplasticity in the motor cortex is a dynamic process that adjusts its functional resources to novel motor demands and altered connectivity. Neuroplasticity after stroke seems to utilize the remaining networks of the affected hemisphere and in homologous regions of the unaffected hemisphere. This may maximize the recovery of neural function or reorganize remaining intact synaptic connections to assist in compensatory adjustments [109]. Functional improvement can result from either structural regeneration in the impaired hemisphere which possibly gives rise to restoration of the original function [111] or plastic rearrangements of intact fibers to facilitate compensatory strategies. The latter process is presumably associated with the development of compensatory, new movement trajectories that differ from original performance [112]. Although the causal role of tDCS in neuroplasticity has not yet been elucidated completely, an increasing body of evidence demonstrates its usefulness as a therapeutic procedure to promote motor and cognitive recovery after stroke [104]. For example, both cortical plasticity and corticomotor excitability can be enhanced by anodal tDCS [40, 113]. It has also been shown that new motor cortex neurons can be recruited to directly control muscle activity [114]. The following summarizes the recent advancement of tDCS in the treatment of stroke-induced motor and cognitive dysfunction.

## 5. tDCS to Ameliorate Poststroke Motor Symptoms 

The three major mechanisms of neurophysiological effects induced by tDCS include (a) improvements in regional cerebral blood flow (rCBF) [115], (b) facilitation of synaptic efficacy [86, 116], and (c) expression of neurotrophic factors [40]. Specifically, the finding of altered neurotrophic factor expression opened a new chapter for a wide range of experimental and clinical applications for stroke-induced motor symptoms.

Some of the most disabling aspects of stroke involve mild to severe sensorimotor impairments [117]. The patterns of sensorimotor improvement during the subacute and chronic phases suggest different roles for the ipsilesional (affected) and contralesional (unaffected) hemispheres. It has been reported that the enhanced neural activity of the contralesional motor areas prevents recovery of motor impairments in the subacute phase [118]. This detrimental effect is mainly caused by the overactivity of the contralesional hemisphere, which transfers to beneficial consequences for the affected hand in the chronic phase after stroke. These findings demonstrate the dynamic interplay of both hemispheres throughout the time course of recovery [119], supporting the hypothesis that the unaffected hemisphere contributes to compensation of functional impairments in the affected hemisphere [104].

## 6. Use of Anodal tDCS after Stroke

A number of studies have investigated the consequences of anodal tDCS in stroke recovery [120]. Focusing on motor performance (finger acceleration measurement and Box and Block Test (BBT)) in subacute poststroke patients, Kim et al. [22] applied anodal tDCS to the ipsilesional cortical region of ten subacute stroke patients approximately 12 weeks after the infarct. In these patients anodal tDCS significantly improved motor performance with an interesting poststimulation effect. While finger acceleration remained enhanced for 30 min after stimulation, enhancement in BBT performance was maintained for 60 min. Using a different tool (Jebsen-Taylor Hand Function Test (JTT)) to evaluate poststimulation motor performance in stroke patients, similar results for a post-stimulation time course were obtained by Fregni et al. [23] and Hummel et al. [108]. Such improvements of motor function by anodal tDCS alone can last for two weeks after treatment [121].

Anodal tDCS also induces a characteristic therapeutic profile as a function of poststroke time interval. Notably, anodal tDCS may have greater therapeutic benefit if not applied immediately after the infarct. A recent study showed that the degree of functional improvement after an ischemic lesion in rats was greater when anodal tDCS was applied one week rather than one day after the injury [122]. This suggests that late application of anodal tDCS may enhance poststroke neural reorganization including synaptic plasticity, possibly via reduction in contralesional inhibitory loads on the ipsilesional hemisphere.

One interesting feature of anodal tDCS on motor imagery has been reported recently [123]. This study investigated the effect of anodal tDCS on desynchronization of alpha-band (mu ERD) electroencephalography (EEG) induced by motor imagery in a patient with severe left hemiparetic stroke. Since it is often difficult for patients with severe hemiparesis to produce sufficiently strong mu ERD to activate the brain-machine interface (BMI), anodal tDCS was applied to modulate mu ERD during the motor imagery therapy. The subject received anodal tDCS for five days during finger flexion imagery, which induced a significant increase of ERD recorded over the affected M1 [123]. In spite of the limited sample size in this study, the result indicates that anodal tDCS is capable of improving motor imagery for severely incapacitated stroke patients. Similar findings were reported by Cicinelli et al. [124] using focal transcranial magnetic stimulation in hemiparetic stroke patients. This group demonstrated that motor imagery significantly enhanced the cortical excitability of the hemisphere affected by stroke in a post-acute stage.

Interestingly, increased cortical activity induced by anodal stimulation bears similarity to an activated (attentive-like) brain state [125]. Thus, one might argue that increasing neuronal activity with anodal stimulation may emulate the effect of attention. This idea is consistent with other studies in which the application of anodal tDCS had a striking resemblance to the effects of increased attention. For example, anodal tDCS can improve the working memory when applied over the prefrontal cortex [126], facilitate problem solving when applied over the temporal lobe [127], and improve motor performance when applied over the motor cortex [25, 93]. Although the proposed idea linking the effect of anodal tDCS to enhanced attention is speculative, it nevertheless is noteworthy because this concept may lead to better understanding of the mechanisms of tDCS and potential therapeutic avenues. One may thus argue that the therapeutic effect of tDCS when combined with physical rehabilitation may work by helping motor networks to fine-tune and “pay attention” to an exercise, thereby enhancing its efficacy.

## 7. Use of Cathodal tDCS after Stroke

The inhibition of cortical excitability in the contralesional hemisphere also promotes motor recovery in stroke patients. This suggests that the therapeutic effectiveness of tDCS extends beyond the application of anodal tDCS over the ipsilesional motor cortex. It has been shown that cathodal tDCS reduces contralateral excitability or inhibitory signals from the contralesional to ipsilesional hemisphere [23, 121, 128]. It has also been reported that the contralesional hemisphere exerts a constant inhibitory drive over the ipsilesional hemisphere in the generation of voluntary movements [119, 129]. The magnitude of this inhibition correlates with the degree of motor impairment after stroke.

A number of studies have investigated the changes in interhemispheric interactions by cathodal tDCS. A recent study assessed the effect of cathodal stimulation on the contralesional motor cortex in 12 well-recovered chronic patients with subcortical stroke [130]. They were tested on the acquisition and retention of complex sequential finger movements of the paretic hand [130]. The authors reported that after only two training sessions with cathodal tDCS, the acquisition of a new motor skill was facilitated compared with sham stimulation [131]. Moreover, the motor improvement was significantly correlated with the changes of tDCS-induced intracortical inhibition [131]. Even in healthy subjects, cathodal tDCS was shown to enhance selective muscle activation of the ipsilateral biceps brachii (BB, antagonist) in a task-specific manner [132]. Furthermore, abnormal movements can be induced by failure of suppression of antagonist muscles, a process which is often observed in proximal upper limbs of stroke patients. Since cathodal tDCS can selectively activate muscles in the proximal upper limb area of the ipsilateral M1, it is expected to aid poststroke motor rehabilitation in these patients [132]. Despite its benefits, the effects of cathodal tDCS appear to be case sensitive, and therefore, the stimulation protocol needs to be tailored to the needs of individual patients [133]. For example, cathodal tDCS improved selective proximal upper limb control only for mildly impaired stroke patients but worsens it for moderate to severely impaired patients [133].

Several studies have also compared the effectiveness of cathodal tDCS and anodal tDCS on stroke patients and healthy subjects [6, 67, 134, 135]. It is difficult to draw a definite conclusion; however, based on the findings that both anodal and cathodal tDCS have beneficial effects, it is reasonable to expect that simultaneous application of cathode and anodal tDCS together with physical therapy enhance the therapeutic efficacy of tDCS. 

## 8. Combined Anodal and Cathodal tDCS Approach

It has been shown that greater and longer-lasting effects can be achieved by bihemispheric tDCS, where an anode is positioned over the affected region, and the cathode is located in the opposite hemisphere [24, 113, 120, 130, 136, 137]. For example, a sham-controlled study investigated the efficiency of the bihemispheric tDCS with simultaneous physical and occupational therapy [138]. Stroke patients with residual moderate to severe hemiparesis received five consecutive sessions of the combined intervention. The authors reported greater functional improvement than the sham group [138]. In addition, the effects persisted for at least one week after the treatment was discontinued. Recently, these authors also examined the effects of two 5-day intervention periods of bihemispheric tDCS and simultaneous occupational and physical therapy [130]. They found that the second 5-day session resulted in additional functional improvement though the gain was lower than the first 5-day session. These studies suggest that the bihemispheric tDCS application combined with physical therapy may be an ideal strategy to generate long-lasting functional improvement. Although the exact mechanisms of behavioral improvement after bihemispheric tDCS are still not well understood, it was suggested that due to “competition” between hemispheres, the contralesional hemisphere has mostly an inhibitory influence on stroke affected area. Thus, downregulating activity in the contralesional hemisphere with a cathode may aid recovery of the affected hemisphere. Transient hyperexcitability of the hemisphere contralateral to the neocortical infarction has been documented [33, 61, 139, 140] and is considered as one of the major causes of poststroke recovery linked with plastic reorganization. Such interactions are reflected in a study performed by Traversa et al. [141]. They reported that between 2 and 4 months following a monohemispheric stroke, the motor output is still undergoing a remarkable reorganization. This reorganization is characterised by the enlargement of the output area, increased motor-evoked potentials amplitude on the affected hemisphere, and a larger than normal contracted motor-evoked potential amplitude from the unaffected hemisphere. Such results reflect transcallosal influences that are linked to clinical outcome. Nevertheless, future research is needed to better understand interactions between cortical hemispheres and how those mechanisms could be used to optimize protocols for tDCS and physical therapies. 

## 9. tDCS and Poststroke Cognitive Symptoms 

We have so far reviewed the effects of tDCS focusing on motor function. It has been shown that tDCS is also effective for facilitating language processing and articulation, which are other major complications after stroke [136, 137, 142, 143]. Imaging studies suggest that spontaneous language recovery in stroke patients and greater activation of the left hemisphere regions are positively correlated [144–146]. Increased left hemisphere activity of stroke patients with aphasia was also associated with greater naming accuracy [147]. Thus, it was proposed that intact areas of the left hemisphere may be suitable tDCS targets to promote recovery from aphasia. A well-controlled study tested this hypothesis and revealed that anodal tDCS over the left frontal cortex can lead to enhanced naming accuracy [148]. The improvement persisted for at least one week after the application of the tDCS. Interestingly, the cathodal tDCS over the right healthy Broca's homologue area also improved performance in a picture naming task in aphasic patients who had suffered unilateral stroke [128]. 

Similar effects of tDCS on language ability were also reported [149]. Patients with stroke-induced aphasia were given five consecutive daily sessions of tDCS. The stimulation targeted Wernicke's area while the patients performed a picture-naming task. The results indicated that tDCS significantly improved the patients' accuracy in picture naming and reduced the naming latencies. Taken together, these studies demonstrate that tDCS may provide an effective supplementary therapy for anomia in stroke patients.

## 10. Limitations of tDCS

Because evaluation of tDCS is being conducted mainly in academia, studies are not widely standardized regarding variables and population samples, therefore limiting generality of conclusions [150]. These findings are also limited by small sample sizes and experimental design. Although animal studies are useful for exploring physiological aspects of tDCS mechanisms, differences in cortical architecture as compared to humans may pose problems in translating findings from animal research to humans (i.e., positioning of electrodes, stimulation parameters, etc.). Thus, despite multiple studies showing benefits of tDCS, the jury is still out whatever these results will translate into real-world benefits [151]. 

## 11. Future Directions and Synthesis: Optimizing tDCS Based on Brain Synchrony

As we learn more about the effects of tDCS on stroke outcome, it should become possible to optimize parameters of tDCS to maximize its effectiveness. For example, the optimal duration of stimulation, current intensity, and the effects of direct current or additional temporal modulation to enhance therapy effectiveness still remains to be determined. Furthermore, critical variables for tDCS outcomes include the location and the number of electrodes, the polarity of each electrode, and how all those parameters should be adjusted for each individual patient. Correlational observations of tDCS parameters and outcomes in brain activity may aid in developing universal guidelines for tDCS stimulation protocols. The guidelines are expected to be applicable to not only stroke but also other neurological conditions. Thus, it may help to use specific features of brain activation as biomarkers to determine tDCS efficacy. 

Since motor or cognitive milestones in functional recovery may show considerable variation between individual stroke patients, it may be more suitable to choose electrophysiological variables as an instantaneous and a meaningful measure of tDCS treatment success. Based on multiple lines of evidences described below, we hypothesize that one of such biomarkers could be a synchrony between cortical areas. We envision that in the future, the effectiveness of tDCS in stroke therapy may be optimized by applying electrical stimulation with parameters that maximize synchronous communication among cortical areas.

**Figure 3:**
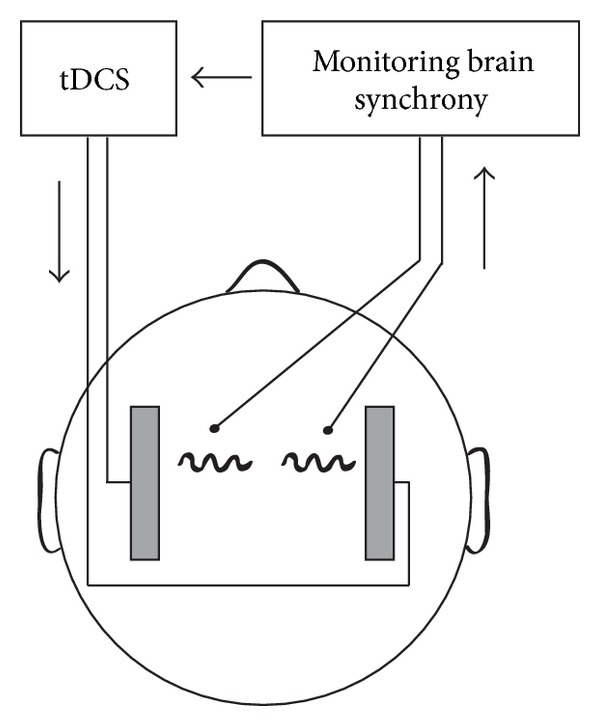
Using brain activity as a “biomarker”. We hypothesize that choosing tDCS parameters to maximize synchrony between cortical areas could lead to improved communication between the affected areas and thus result in more effective stroke rehabilitation.

The support for the notion that cortical synchrony could be a useful biomarker comes from a number of studies, showing that a decrease in synchrony relates to a general reduction in connectivity among brain areas following stroke. Using data from diffusion magnetic resonance imaging, Crofts and Higham [152] compared connectivity between 56 brain areas in stroke patients and healthy controls and showed decreased communication among a number of brain regions in stroke patients. Similar results were obtained using near-infrared spectroscopy [153], showing that the interhemispheric correlation coefficient was reduced in stroke patients. Consistent with these results is the reduction in interhemispheric functional connectivity in a rat model of stroke using low-frequency BOLD fluctuations in MRI [154]. This reduction was also correlated with decreased sensorimotor function [154]. Similarly, analysis of functional connectivity with EEG also revealed reduced interhemispheric coherence in stroke patients [155, 156]. An interesting interpretation of this decline in interactions among brain areas after stroke was recently suggested based on the analysis of the topological configuration of the resting-state networks [157]. The authors concluded that brain networks shift toward a more random and less optimized mode of function after stroke [157]. Thus, decreased coherence between hemispheres of various brain areas may reflect reduced efficiency of communication among neuronal networks after stroke. To reduce this feature and promote recovery, we hypothesize that the application of tDCS with stimulation parameters adjusted online to increase coherence between affected areas (Figure 3) could result in maximizing therapeutic outcome. Nevertheless, this approach will require special safety considerations as stroke patients are more prone to seizures [158].

In conclusion, tDCS represents a promising technique that can improve stroke rehabilitation by modulating neuronal activity and by promoting neuronal plasticity. We suggest that an interesting future direction will involve using cortical synchrony as a “biomarker” of tDCS efficacy which will help to develop more effective and reliable guidelines for the application of tDCS in stroke therapy.
